# Rapid and deep-scale ubiquitylation profiling for biology and translational research

**DOI:** 10.1038/s41467-019-14175-1

**Published:** 2020-01-17

**Authors:** Namrata D. Udeshi, Deepak C. Mani, Shankha Satpathy, Shaunt Fereshetian, Jessica A. Gasser, Tanya Svinkina, Meagan E. Olive, Benjamin L. Ebert, Philipp Mertins, Steven A. Carr

**Affiliations:** 1 Broad Institute of MIT and Harvard, Cambridge, MA 02142 USA; 2 Division of Hematology, Brigham and Women’s Hospital, Boston, MA 02115 USA; 3 Department of Medical Oncology, Dana-Farber Cancer Institute, Boston, MA 02215 USA; 4 Howard Hughes Medical Institute, Dana-Farber Cancer Institute, Boston, MA 02215 USA; 5 Max Delbrück Center for Molecular Medicine in the Helmholtz Society, Berlin, Germany; 6 Berlin Institute of Health, Berlin, Germany

**Keywords:** Proteomics, Mass spectrometry, Ubiquitylation

## Abstract

Protein ubiquitylation is involved in a plethora of cellular processes. While antibodies directed at ubiquitin remnants (K-ɛ-GG) have improved the ability to monitor ubiquitylation using mass spectrometry, methods for highly multiplexed measurement of ubiquitylation in tissues and primary cells using sub-milligram amounts of sample remains a challenge. Here, we present a highly sensitive, rapid and multiplexed protocol termed UbiFast for quantifying ~10,000 ubiquitylation sites from as little as 500 μg peptide per sample from cells or tissue in a TMT10plex in ca. 5 h. High-field Asymmetric Waveform Ion Mobility Spectrometry (FAIMS) is used to improve quantitative accuracy for posttranslational modification analysis. We use the approach to rediscover substrates of the E3 ligase targeting drug lenalidomide and to identify proteins modulated by ubiquitylation in models of basal and luminal human breast cancer. The sensitivity and speed of the UbiFast method makes it suitable for large-scale studies in primary tissue samples.

## Abstract (web summary)

Comprehensive protein ubiquitylation profiling by mass spectrometry typically requires large sample amounts, limiting its applicability to tissue samples. Here, the authors present an optimized proteomics method that enables multiplexed ubiquitylome analysis of cells and tumor tissue samples.

## Introduction

The post-translational biological process of ubiquitylation attaches the small protein ubiquitin to substrate proteins through the action of a highly coordinated cascade of activating (E1), conjugating (E2), and ligating (E3) enzymes believed to number in excess of 600 (refs. ^1,2^). Ubiquitin is linked to substrate proteins via its C terminus that forms an isopeptide bond most often with the epsilon amino group of lysine residues in the other proteins. Like protein phosphorylation, ubiquitylation is reversible, a process governed by yet another set of over 100 enzymes termed deubiquitinases^3,4^. The E3 ligases attach ubiquitin chains to specific substrate proteins, thereby regulating a wide variety of biological processes, including protein degradation, modulation of substrate activity, and progression through the cell cycle. Mutations and other changes that result in dysregulation of either ligases or deubiquitinases may lead to aberrant activation or deactivation of pathways involved in many disease processes, notably cancer progression and metastasis, immune disorders, and neurological diseases among others. Their role in oncogenesis has been well-described^4–8^. The potential druggability of E3 ligases with small molecules such as lenalidomide as treatments for a variety of cancers has greatly increased interest by the biotechnology and pharmaceutical industries in this class of enzymes^6,9^.

**Fig. 1:**
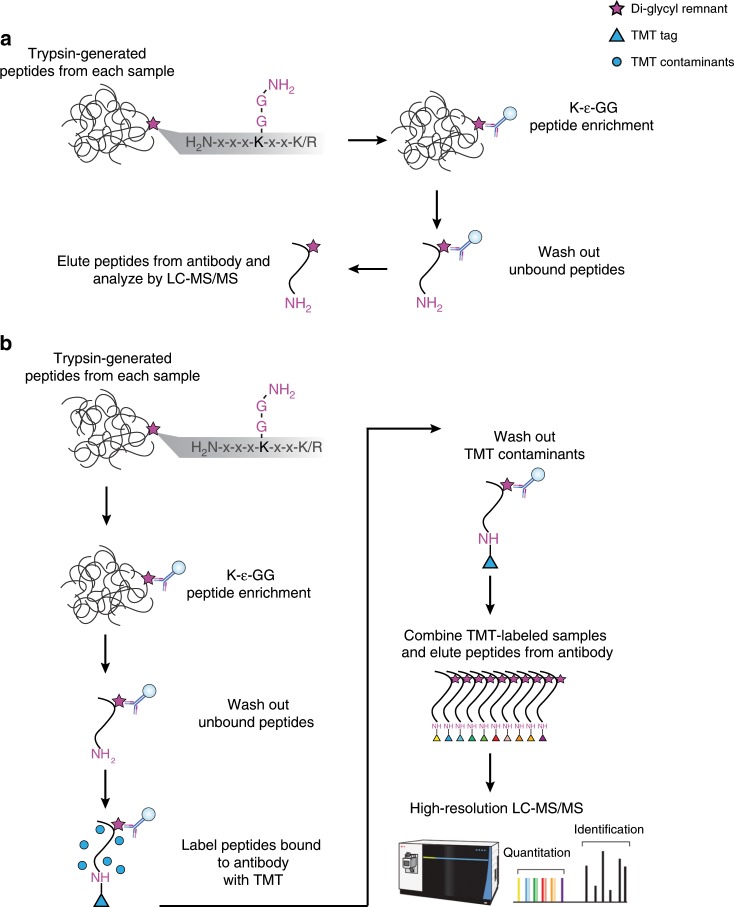
Comparison of methods for multiplexed ubiquitylome analysis. **a** Conventional workflow for an anti-K-ɛ-GG antibody-based enrichment without isobaric labeling, where the antibody recognizes primary amine containing K-ɛ-GG epitope. **b** UbiFast workflow employing on-antibody labeling with isobaric mass tag reagents (e.g., TMT10/11) for anti-K-ɛ-GG antibody-based enrichment of ubiquitylated peptides.

While liquid chromatography-mass spectrometry (LC-MS/MS) is the leading method for unbiased analysis of protein modifications, comprehensive profiling of endogenous ubiquitylation sites has, until recently, been very difficult^10^. The reasons for this include the large size of the modification (molecular mass of 8.6 kDa), the presence of polyubiquitylated modifications, and the low stoichiometry of ubiquitylation^1,2^. The enzyme trypsin is generally used to generate peptides suitable for proteome analysis by LC-MS/MS. Proteolysis of ubiquitylated proteins with trypsin cleaves backbone arginine (Arg) and lysine (Lys) residues in the substrate protein as well as in the attached ubiquitin; this process creates tryptic peptides in which the C-terminal Gly–Gly dipeptide of ubiquitin is still attached to the side chain of Lys residues (Fig. 1a). Importantly, the presence of this side-chain modification on Lys prevents cleavage at that site by trypsin, thus producing a tryptic peptide with an internal modified Lys residue. Development and commercialization of antibodies that recognize this di-glycyl remnant (K-ɛ-GG) and enrich these formerly ubiquitylated peptides was the breakthrough that made comprehensive profiling of ubiquitylation sites by LC-MS/MS possible (Fig. 1a)^10–13^.

To enable precise relative quantification of ubiquitylated peptides and sites across differing samples under perturbation conditions, SILAC (Stable Isotope Labeling by Amino Acids in Cell Culture) has been historically used to differentially label cells grown in culture prior to antibody enrichment. SILAC enables comparison of ubiquitylation sites from up to three samples in a single experiment. We and others have successfully applied this approach in a range of quantitative biological studies using metabolically labeled cells^6,10,14–18^.

Isobaric chemical tags, such as the Tandem Mass Tag (TMT) system, offer many advantages over SILAC for quantifying post-translational modifications by mass spectrometry (MS). They facilitate a decrease in starting material used for enrichment, enable comparison of 11 or more conditions in a single experiment, and significantly minimize the number of missing peptides detected and quantified across all experimental conditions relative to label-free or SILAC-based experiments. However, a major limitation of the ubiquitylation profiling approach described above has been that the commercially available antibodies used to recognize and enrich peptides having the di-glycyl remnant on the side-chain of lysine do not work when the N-terminus of the di-glycyl remnant is derivatized with either iTRAQ or TMT. Efforts to produce antibodies that selectively enrich either an iTRAQ- or TMT-derivatized K-ɛ-GG peptide and not other iTRAQ or TMT-labeled peptides have failed to date (unpublished). Until recently this limitation has restricted use of ubiquitin profiling to fast growing cell lines that can be metabolically labeled in culture and has prevented multiplexed, quantitative analysis of human or animal-derived tissues or primary cell culture models using isobaric reagents.

To address the need for profiling ubiquitylation sites in tissue samples, Rose et al.^19^ introduced a method where samples are enriched at the peptide level using the anti-K-ɛ-GG antibody prior to labeling with TMT10 reagents. After elution from the antibody and labeling, the enriched peptides are subjected to high pH reversed-phase chromatography using spin columns, fractionated into six fractions, and analyzed by LC-SPS-MS3. In this study, 5000–9000 ubiquitylated peptides were quantified in cells using 1 mg sample/TMT label state and in tissue using 7 mg of sample/TMT label state using 18 h of instrument time. This approach represented a breakthrough in the ability to employ isobaric chemical tagging for ubiquitylation profiling of tissues. However, the large amount of sample and lengthy analysis time required to achieve significant depth of detection and quantification of the ubiquitylome would likely preclude the use of this approach for analysis of more limited samples such as primary cells and human tumor samples. These limitations led us to consider other possible approaches to overcome the inability to use anti-K-ɛ-GG antibodies with TMT labeling. MS has previously been used to map the linear amino acid epitope of a protein recognized by an antibody (Ab)^20,21^. In these studies, protein in free and antibody-bound forms was proteolytically digested and the resulting peptides analyzed by MS. The epitope was protected from proteolysis when the antibody was bound and could be identified on the basis of the differential MS analysis. Inspired by this earlier work, we hypothesized that the di-glycyl remnant of ubiquitylated peptides would not be solvent exposed when bound to the anti-K-ɛ-GG antibody.

Leveraging this approach, we here develop a method that increases both sensitivity and throughput for highly multiplexed ubiquitylation profiling. In this approach which we term UbiFast, K-ɛ-GG peptides are labeled with TMT reagents while still bound to the anti-K-ɛ-GG antibody. By doing so, the amine-reactive, NHS-ester group of the TMT reagent reacts with the peptide N-terminal amine group and the ε-amine groups of lysine residues, but not the primary amine of the di-glycyl remnant. TMT-labeled K-ɛ-GG peptides from each sample are combined, eluted from the antibody, and analyzed by single-shot, high-performance LC-MS/MS **(**Fig. 1b). We reason that TMT labeling of K-ɛ-GG peptides bound to the antibody beads could potentially increase sensitivity, reduce the levels of TMT-based contaminant side-products, and avoid the need to offline fractionate prior to MS measurement. We use this method to profile patient-derived breast cancer xenograft tissue samples in a TMT10-plex experiment using 0.5 mg input/sample to quantify >10,000 distinct ubiquitylation sites. The method paves the way for quantification of ubiquitin remnants in human tissues and more physiological patient-derived cell models where sample amounts are limiting.

## Results

### Optimization of on-antibody TMT labeling

To establish the feasibility of labeling peptides with TMT reagents while bound to the anti-K-ɛ-GG antibody, and to determine the optimal amount of labeling reagent and labeling time, K-ɛ-GG peptides were enriched in triplicate from 1 mg of Jurkat peptides cells and labeled with varying amounts of a single TMT reagent for varying durations while peptides were still bound to antibody (Fig. 1b, Supplementary Fig. 1, Supplementary Data 1 and 2). We found that 10 min labeling with 0.4 mg of TMT reagent provided the best balance of numbers of identified TMT-labeled K-ɛ-GG peptides and completeness of labeling (>92%) for K-ɛ-GG peptides bound to anti-K-ɛ-GG antibody. In order to prevent potential TMT cross-labeling when samples are combined, it is important that the labeling reaction be fully quenched. Therefore, we tested quenching of the TMT reactions with 5% hydroxylamine. The quenching was successful in stopping the labeling reaction, evidenced by a slightly reduced labeling efficiency. Importantly, quenching also increased the number of K-ɛ-GG peptides identified by almost 10% (Supplementary Fig. 2, Supplementary Data 3a). We find that label-free analysis of enriched K-ɛ-GG peptides results in lower relative yield of K-ɛ-GG peptides when compared to K-ɛ-GG enrichment coupled to TMT labeling (Supplementary Fig. 2, Supplementary Data 3b).

### Comparison of on-antibody and in-solution TMT labeling

We next compared in-solution TMT labeling of K-ɛ-GG peptides to on-antibody TMT labeling of K-ɛ-GG peptides with respect to the total numbers of K-ɛ-GG peptides detected, the relative yield of K-ɛ-GG peptides (relative yield is the percentage of K-ɛ-GG peptides relative to the total peptides identified in the sample) and the efficiency of TMT labeling (Supplementary Fig. 3a–d, respectively). Jurkat peptide samples (1 mg, each) were enriched with the anti-K-ɛ-GG antibody and peptides were labeled with a single TMT reagent, either while the peptides were bound to the antibody or using the in-solution labeling method previously described^19^ (Supplementary Fig. 3a). On-antibody TMT-labeled samples resulted in 6087 K-ɛ-GG PSMs with a relative yield of 85.7%, while samples labeled in-solution resulted in 1255 K-ɛ-GG PSMs with a relative yield of 44.2% (Supplementary Fig. 3b, c, Supplementary Data 4). The labeling efficiency of on-antibody and in-solution TMT labeling methods were both high, with 98% of peptides being at least partially labeled (Supplementary Fig. 3d).

As both labeling methods are designed to be used with multiplexed samples, we carried out a head-to-head comparison where both labeling methods were used to analyze 10 process replicates of peptides from HeLa cells, with 1 mg peptide input per replicate (Supplementary Fig. 4) using single-shot LC-MS/MS methods with two injections per sample and longer gradients than in the initial experiments described above (154 vs. 110 min/injection) totaling to 5.1 h of instrument time. We find that injecting samples twice leads to a moderate boost in identifications. In concordance with the single sample experiments, the on-antibody labeling method identified many more fully quantified, distinct K-ɛ-GG peptides compared to the in-solution labeling method (9069 vs. 4587 peptides) (Supplementary Fig. 4b, Supplementary Data 5a, b). The relative yield of K-ɛ-GG peptides was 85.4% using the on-antibody labeling approach compared to 49.9% for the in-solution labeling method (Supplementary Fig. 4b). There was a significant overlap in the commonly identified sites, with approximately 80% of the sites identified using the in-solution labeling method also identified by the on-antibody labeling approach (Supplementary Fig. 4c). The median CVs between the 10 process replicates were very similar for both TMT labeling methods (Supplementary Fig. 4d). TMT labeling of K-ɛ-GG peptides bound to the antibody beads could also potentially reduce the levels of TMT-based contaminant side-products in the final sample, which often show up as +1 precursors^22^, and avoid the need to offline fractionate prior to MS measurement. Singly charged precursors constituted a far lower percent of the total precursor ion current using on-antibody TMT labeling than in-solution labeling (3.9% vs. 16.5% (Supplementary Fig. 4e, Supplementary Data 5c, d). These results demonstrate that on-antibody TMT labeling more effectively removes TMT contaminants and improves K-ɛ-GG enrichment specificity which is what likely leads to an increase in identified and fully quantified K-ɛ-GG peptides.

### Identification of lenalidomide targets in multiple myeloma

**Fig. 2:**
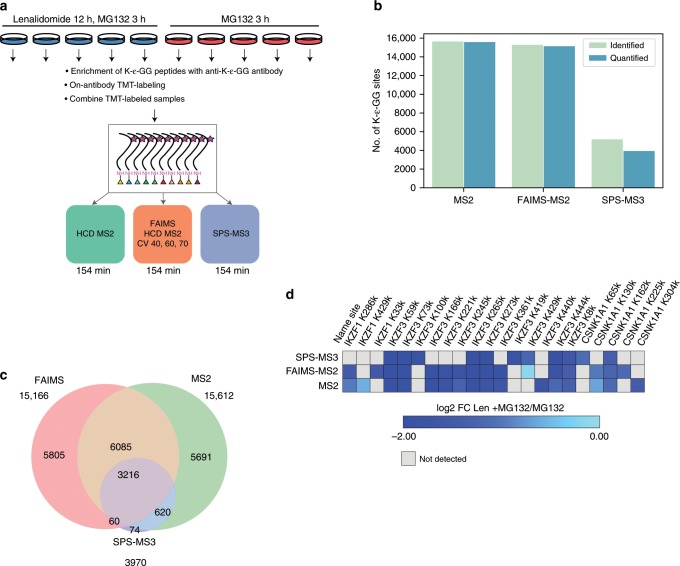
Benchmarking UbiFast. **a** Experimental design for TMT10-based assessment of global changes in ubiquitylation and protein levels. On-antibody K-ɛ-GG enrichment and TMT labeling are benchmarked in lenalidomide-treated MM1S cells using HCD-MS2, SPS-MS3, and FAIMS-MS2. **b** Bar plots show the number of distinct quantified K-ɛ-GG sites identified in each type of MS experiment. **c** Venn diagram shows the overlap of quantified K-ɛ-GG sites across each MS experiment type (HCD-MS2, SPS-MS3, FAIMS-MS2). **d** Heatmap of log2 fold-change values for 12 h lenalidomide +3 h MG-132 vs. 3 h MG-132 treated MM1S cells for IKZF1, IKZF3, and CSNK1A1. HCD-MS2 and FAIMS-MS2 data have been filtered to show only those sites that had >90% precursor isolation purity.

In prior work, we employed ubiquitylation profiling by MS in SILAC-labeled multiple myeloma cell lines to reveal the mechanism of action of lenalidomide, an anti-tumor drug in multiple myeloma^6^. This study, which utilized 10 mg of peptide/SILAC state, revealed that lenalidomide causes degradation of the Ikaros transcription factors IKZF1 and IKZF3. To benchmark our approach, we repeated our previously published SILAC-based experiment by treating MM1S cells with 1 μM lenalidomide for 12 h and MG-132 for 3 h, or with only MG-132 for 3 h using either HCD-MS2, SPS-MS3 ^22,23^, or FAIMS-MS2, a method recently shown to increase the numbers of detected and quantified peptides in proteome studies^24,25^ (Fig. 2). We then enriched K-ɛ-GG peptides and carried out on-antibody TMT10 labeling for ubiquitylome profiling as described above using just 1 mg of peptide input per sample. The final enriched TMT10-plex sample mixture was divided into thirds and one-third of the sample was analyzed by each of the three data acquisition methods (Fig. 2a). Using the MS2 approach we quantified 15,612 distinct ubiquitylation sites (Fig. 2b, c, Supplementary Data 6a). Using FAIMS-MS2, the number of quantified ubiquitylated peptides was similar (15,166) (Supplementary Data 6a). In contrast, only 3970 ubiquitylated peptides were observed using SPS-MS3 (Supplementary Data 6a), more than threefold fewer than using either MS2 or FAIMS-MS2. The reproducibility of all three methods was high, with median coefficients of variation across process replicates of <20% (MS2: 13.2%, FAIMS-MS2:15.8%, SPS-MS3: 10%).

Using either MS2 or FAIMS-MS2, we observed the expected changes in ubiquitylation induced by lenalidomide treatment on multiple lysine residues of IKZF1, IKZF3, and CSNK1A1. Because IKZF1 and IKZF3 are rapidly ubiquitylated and targeted for degradation, the resulting decrease in corresponding ubiquitylated peptides is a result of decreased absolute levels of these proteins^6^. However, using SPS-MS3, changes in ubiquitylation of one of the key transcription factors, IKZF1, were not detected, and fewer ubiquitylated peptides overall were detected on the IKZF3 and CSNK1A1 (Fig. 2d). Importantly, the expected ubiquitylated and degraded proteins were identified using only 1 mg peptide/sample compared to a total of 10 mg input/sample used in the SILAC approach^6^. Together, these results support the feasibility and value of multiplexed ubiquitylation profiling in systems where sample amounts are limiting.

### Accuracy of ubiquitylated peptide quantification

Using HCD-MS2 we observed several ubiquitylated peptides from IKZF1, IKZF3, and CSNK1A1 that appeared to not be significantly down-regulated upon lenalidomide treatment despite the observation of other ubiquitylated peptides from these same proteins that were strongly regulated (Supplementary Fig. 5a). We reasoned that this was most likely the result of ratio compression, an expected and well-described issue related to TMT quantitation^22,26^. Precursor isolation purity filtering is a common approach for removing interferences in TMT experiments^27,28^. Filtering MS2 (Supplementary Fig. 5b, Supplementary Data 6b) and FAIMS-MS2 data (Supplementary Data 6b) for only those peptides having >90% precursor isolation purity (PIP) significantly improved quantification accuracy while decreasing the total number of ubiquitylated peptides observed in MS2 by 40.8% (Supplementary Data 6b). As expected, FAIMS-MS2 reduced precursor co-isolation interference and stringent PIP filtering retained relatively more ubiquitylated peptides than stringent PIP filtering of MS2 data, reducing the total number of ubiquitylated peptides by only 28% (Supplementary Fig. 5c, Supplementary Data 6b). SPS-MS3 had the greatest accuracy, but identified fewer of the statistically significant sites that were seen by either MS2 and FAIMS-MS2 (Supplementary Fig. 5d, Supplementary Data 6).

### Applying UbiFast for rapid profiling of tumor tissue

**Fig. 3:**
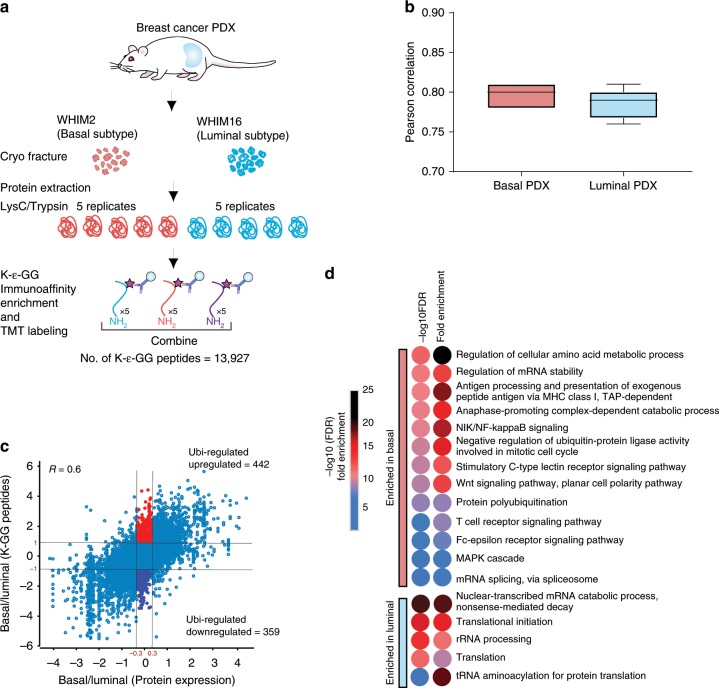
Ubiquitylation analysis of breast cancer PDX models. **a** Schematic diagram showing experimental design used to enrich di-Gly modified peptides from the Luminal and Basal breast cancer PDX models. **b** Pearson correlation between Luminal and Basal PDX replicates (*n* = 5 process replicates). Boxplot depicts upper and lower quartiles, with median shown as a solid line. Whiskers show 1.5 interquartile range. Source Data are provided in Supplementary Data 7a. **c** Scatter plot showing log2 fold-change between Basal and Luminal PDXs, for expression of ubiquitylated sites and the corresponding protein. The proteins that were upregulated or down-regulated exclusively at the level of their ubiquitylation are indicated by red or blue points, respectively. **d** Pathway enrichment of proteins regulated primarily at the level of ubiquitylation.

To demonstrate the utility of the UbiFast approach to quantitatively profile the ubiquitylome of small amounts of tissue, we isolated tumors from two previously described breast cancer patient-derived xenograft (PDX) models, representing basal (WHIM2) and luminal (WHIM16) subtypes of breast cancer, respectively^29^ (Fig. 3a). These models faithfully reproduce genomic features of the disease, exhibiting distinct expression of key basal and luminal genes, and proteome-driven basal and luminal pathway signatures^30^. Ubiquitylated peptides were enriched from five replicates each of WHIM2 and WHIM16 using 0.5 mg of peptide per sample. As described above, peptides enriched using anti-K-ɛ-GG antibody were TMT labeled on antibody. TMT-labeled peptides from each sample were then combined and analyzed using two single-shot LC-MS/MS runs. FAIMS was used with two compensation voltage (CV) sets per injection (see Methods) to reduce ratio compression and increase the total number of ubiquitylated peptides identified. We find that injecting samples twice using a different compensation voltage set for each analysis results in lower peptide overlap (and therefore higher total identifications) relative to two independent injections without FAIMS. A total of 13,501 human K-ɛ-GG peptides were observed, 81% (10,942) of which were quantified across all 10 TMT channels (Supplementary Data 7a). The correlation across replicates was high, with a median Pearson correlation of 0.78–0.81 (Fig. 3b).

To identify the relationship between ubiquitylated peptides and their protein expression, we compared our ubiquitylome dataset to a previously published deep-scale proteomics dataset obtained on the same basal and luminal PDX breast cancer models that used a similar TMT10 design with analysis of 24 offline basic reverse phase fractions (vs. UbiFast using single-shot methods) using a similar TMT10 workflow^31^ (Supplementary Data 7c). There was a high degree of correlation between the fold-changes observed between basal and luminal expression at the level of K-ɛ-GG modified peptides and the corresponding protein abundance changes. This further supports robust quantification of ubiquitylated peptides relative to deep-scale fractionated proteome as it is expected that the majority of changes observed in the ubiquitylome will be driven by changes in the levels of the corresponding proteins (Pearson correlation = 0.6) (Fig. 3c). However, in addition, we also identified a total of 801 K-ɛ-GG peptides that were regulated (log2 fold-change >1 and <−1) primarily at the level of ubiquitylation (referred to as Ubi-regulated), but that did not show regulation at the protein level (log2 fold-change >−0.3 and <0.3) (Fig. 3c, Supplementary Data 7,d). A total of 110 sites showed marked outlier regulation with log2 fold-change >2 and <−2 (Fig. 3c, Supplementary Data 7d). Of note, we observed differential ubiquitylation of 15 E3 ligases; TRAF7, HUWE1, NEDD4L, UBE3A, UBE4B, CBL, BIRC6, RAD18, RNF146, MIB1, HERC2, and AFF4 showed upregulated ubiquitylation in Basal subtype whereas ITCH, TRIM25, RNF185, and IRF2BPL showed upregulated ubiquitylation in Luminal PDX model (Supplementary Data 7,d). Pathway enrichment of Ubi-regulated proteins (Fig. 3d) showed enrichment of several immune-signaling pathways in the basal PDX models consistent with the critical role of ubiquitylation in immune signaling^14,32,33^. Interestingly, Basal or triple-negative breast cancer shows high immunological activity and are attractive candidates for immuno-oncology therapies^34^ and, therefore, upregulated ubiquitylation of immune signaling is consistent with the “immune hot” environment within the basal PDX tumor.

To begin to evaluate the crosstalk between two distinct lysine modifications, we compared differential ubiquitylation and acetylation (K-Ac) in these basal and luminal PDX models (Supplementary Fig. 6, Supplementary Data 7c). Enrichment of K-Ac peptides was performed^35^. TMT-labeled peptides from five replicates each of the basal and luminal PDX models were mixed at an equal ratio, fractionated into four fractions and subjected to immuno-enrichment using an anti-acetyl antibody (PTM-SCAN acetyl-kit; Cell Signaling Technologies). A total of 11,929 acetylated peptides were identified with 10,967 of these identified across all channels. Over 2550 sites showed both acetylation and ubiquitylation with Pearson correlation of 0.45 (Supplementary Fig. 6a, b, Supplementary Data 7d). Interestingly, a small subset of Ubi-regulated sites were also inversely regulated by acetylation suggesting a regulatory crosstalk between ubiquitylation and acetylation (Supplementary Fig. 6c, d). In summary, this study highlights the feasibility of UbiFast to identify ubiquitylation driven pathways in tumors as well as cell lines, and that it can also be used to investigate the crosstalk of lysine ubiquitylation and other lysine modifications such as acetylation as exemplified in this study. Future studies in a larger number of PDX or human tumors are needed to elucidate in vivo functional roles.

## Discussion

A major advantage of TMT-based quantitation is the ability to analyze a variety of sample types including primary tissue. However, to realistically monitor ubiquitylation in human cancer tissue and patient-derived cell culture models where generation of protein input of >1 mg/sample is not feasible, a highly sensitive method that can be successfully employed in the 500 μg–1 mg range is needed. With this in mind, we have developed a method for rapid, sensitive and multiplexed ubiquitylation profiling in cells and tissue using anti-K-ɛ-GG antibodies to enrich ubiquitylated peptides followed by on-antibody TMT labeling. We directly compared on-antibody TMT labeling to conventional in-solution TMT labeling of enriched K-ɛ-GG peptides and demonstrated that TMT labeling of K-ɛ-GG peptides while bound to the antibody significantly increases sensitivity, due in part to a significant reduction in the level of TMT contaminant side-products in the final processed sample. We have used this method to quantify over 11,000 ubiquitylated peptides from as little as 500 μg input using only 5.1 h of instrument time, demonstrating that the method is suitable for large-scale studies in primary tissue samples.

Relative to our previously published SILAC-based method for ubiquitylation profiling, the UbiFast method enables multiplexing of >3x more samples, uses 6–12x less peptide input per treatment condition, requires much less wet lab processing time, and utilizes 5x less LC-MS measurement time^11^. Additionally, we find that on-antibody TMT labeling increases the relative yield of K-ɛ-GG peptides relative to SILAC, label free (Supplementary Fig. 2), and in-solution TMT labeling-based methods. Because of these complexities and inefficiencies in using the SILAC quantification approach for ubiquitylation profiling in cells noted above, studies focused on identifying drug-induced substrates of E3 ubiquitin ligases are being carried out with increasing frequency by proteome profiling only rather than ubiquitin profiling^36,37^. The simplicity and speed of our on-antibody labeling approach opens the door to deep-scale ubiquitylation profiling to more directly identify the substrates of E3 ligases degraded by the proteasome degradation pathway. Combined with proteome profiling, the UbiFast method should facilitate identification of signaling effects of ubiquitylation as well as substrates of proteasomal degradation. The acetylome and ubiquitylome data sets we provide on the breast cancer xenograft samples are a rich source for the community to begin to probe and elucidate signaling crosstalk between acetylation and ubiquitylation.

A current limitation of the UbiFast method is that the depth of quantification appears to be limited to around 10,000 sites/sample when starting with 0.5–1 mg of sample/TMT channel. While this depth-of-coverage is less than what is achieved in SILAC-based experiments, the input amount requirements are lower, the multiplexing capacity is significantly higher, and the method requires no offline fractionation, making it faster and far easier to implement. This is approximately the same depth of coverage achieved using acetylome profiling, but is substantially less than phosphoprofiling that can produce over 35,000 distinct phosphosites/sample in a 10-plex experiment^30^. Extension of our method to higher-plex isobaric labeling methods such as TMTPro (Thermo Fisher) that enables 16-plex analysis should allow further reduction in the amount of sample/channel needed to achieve current depth of analysis. In addition, the quantitative accuracy of the FAIMS-MS2 data generation method we employ is somewhat lower than can be achieved using SPS-MS3, but the lower accuracy is compensated for by the much higher numbers of sites quantified and able to be shown to be differential between states. Future efforts will also be aimed at reducing the amount of labeling reagent required for on-antibody TMT labeling to further reduce the cost of the UbiFast method. Zecha et al.^31^ have recently presented a protocol for TMT labeling that reduces the quantity of required labeling reagent by reducing reaction volumes.

Beyond applications for ubiquitylation profiling, we hypothesize that on-antibody TMT labeling will be a valuable method to increase the throughput and lower the TMT reagent cost for multiplexed analysis of other PTMs requiring antibody-based enrichment such as lysine acetylation and tyrosine phosphorylation. The UbiFast method is also potentially amenable to serial enrichment analysis workflows^38^ which is especially important when sample amounts are limiting. Finally, we envision that the ease and simplicity of the UbiFast method may be transferable to automated robotic sample-handling platforms to facilitate processing of very large numbers of samples for ubiquitylome analyses^39^.

## Methods

### Ethical compliance

PDX models used in this study were approved by the institutional animal care and use committee at Washington University in St. Louis.

### Anti-K-ɛ-GG antibody crosslinking

For all experiments, the PTM-Scan ubiquitin remnant motif (K-ɛ-GG) kit (Cell Signaling Technology, Kit #5562) was crosslinked. Briefly, antibody-bound beads were washed three times with 100 mM sodium borate (pH 9.0) and incubated with 20 mM DMP for 30 min at room temperature. Beads were washed 2× with 200 mM ethanolamine and incubated overnight at 4 °C with 200 mM ethanolamine. Following this incubation, beads were washed 3× with immunoprecipitation (IAP) buffer (50 mM MOPS, pH 7.2, 10 mM sodium phosphate, 50 mM NaCl) and stored at 4 °C at a concentration of 0.5 μg/μL.

### Optimization of TMT reagent amount and labeling time

Jurkat cells, clone E6-1 obtained from ATCC (TIB-152) were cultured in RPMI-1640 media (Invitrogen) supplemented with 10% dialyzed fetal bovine serum (Sigma-Aldrich), penicillin/streptomycin, and l-glutamine (Invitrogen). Cells were lysed for 30 min with ice cold urea lysis buffer containing 8 M urea, 75 mM NaCl, 50 mM Tris-HCl (pH 8.0), 1 mM EDTA, 2 μg/mL Aprotinin (Sigma), 10 μg/mL Leupeptin (Roche), 1 mM PMSF (Sigma). Samples were spun at 20,000 *g* for 10 min and a BCA assay (Thermo Fisher) was used to determine the concentration of protein in each cleared lysate. Lysis buffer was used to equalize the protein concentration of each lysate to 8 mg/mL. Protein disulfide bonds were reduced with 5 mM dithiothreitol (Thermo Fisher) at 25 °C for 45 min. The proteins were alkylated in the dark using 10 mM iodoacetamide at 25 °C for 45 min. Lysates were then diluted 1:4 using 50 mM Tris, pH 8.0, to lower the urea concentration to 2 M. LysC (Wako) was added to each lysate at a 1:50 enzyme-to-substrate ratio and samples were digested at 25 °C for 2 h. Following LysC digestion, Trypsin (Promega) was added at a 1:50 enzyme-to-substrate ratio and the samples were digested at 25 °C overnight. The digestion was quenched with 100% formic acid to reach a volumetric concentration of 1% formic acid. Samples were spun at 5000 *g* for 5 min to remove precipitated urea and then desalted using Sep-Pak C18 columns (Waters, 500 mg WAT043395). Sep-Pak columns were conditioned with 1 × 5 mL 100% acetonitrile (ACN), 1 × 5 mL 50% ACN/0.1% formic acid (FA), and 4 × 5 mL 0.1% trifluoroacetic acid (TFA). Each sample was loaded onto a column and washed with 3 × 5 mL 0.1% TFA and 1 × 5 mL 1% FA. Peptides were eluted off the column with 2 × 3 mL 50% ACN/0.1% FA and dried down. Peptides were resuspended in 3% ACN, 0.1% FA and a BCA assay (Thermo Fisher) was used to determine the concentration of peptide in each sample. Samples were then aliquoted into 1 mg aliquots, dried down and stored at −80 °C.

For each replicate enrichment, 1 mg of Jurkat peptide sample and 31.25 μg of crosslinked anti-K-ɛ-GG antibody was used. Specifically, 31.25 μg of crosslinked anti-K-ɛ-GG antibody from 0.5 μg/μL solution was pipetted into 1.5 mL Eppendorf tubes. one milligram of Jurkat peptide was resuspended in 1.5 mL of IAP buffer and shaken at room temperature (RT) for 5 min. The pH of each Jurkat peptide sample was checked by spotting ~1 μL of solution on pH paper to ensure the pH was ~7. If the pH was acidic, the samples were titrated using 1 M Tris. Peptide samples were then centrifuged at 5000 *g* for 5 min. Jurkat peptides were added to a tube of aliquoted antibody and incubated for 1 h at 4 °C, with gentle end-over-end rotation. After incubation, all enrichments were kept on ice unless being handled. Following incubation, all samples were centrifuged at 2000 r.c.f. for 1 min and then antibody beads were allowed to settle by letting tubes sit for ~10–20 s on ice. The supernatant (IP flowthrough) was removed and the antibody beads were washed with 1.5 mL of ice cold IAP buffer. For washing the beads, after the addition of the wash reagent, the tubes were inverted ~5 times, centrifuged for ~30–60 s at 2000 r.c.f., allowed to sit for ~10–20 s to let the beads settle, and the supernatant was removed. All washes were completed as quickly as possible. A wash with 1.5 mL of ice cold PBS was performed and antibody beads were resuspended in 200 μL of 100 mM HEPES (pH 8.5).

For optimization of on-antibody TMT labeling, TMT reagent amount (data shown in Supplementary Fig. 1a, b) and TMT labeling time were tested (data shown in Supplementary Fig. 1b, c). For experiments testing TMT reagent amount, 0.8 mg, 0.4 mg, or 0.2 mg of a single TMT10 reagent (Thermo Fisher) was resuspended in 10 μL of ACN and added to each enrichment sample, samples were spun down quickly at 2000 r.c.f. for 5–10 s, and samples were incubated at RT for 5 min shaking at 1400 r.p.m. For experiments testing TMT labeling time, 0.4 mg of a single TMT10 reagent was resuspended in 10 μL of ACN and added to each enrichment sample, samples were spun down quickly at 2000 r.c.f. for 5–10 s, and samples were incubated at RT for 5, 10, or 20 min shaking at 1400 r.p.m.

Following the TMT incubation step, samples were washed with 1.3 mL of ice cold IAP buffer followed by 1.5 mL ice cold IAP buffer, and 1.5 mL of ice cold PBS. The final wash buffer was removed and the enriched peptides were eluted from the antibody beads by adding 150 μL of 0.15% TFA to the beads forcefully enough to resuspend them and incubating for ~5 min at RT. While the beads were incubating in elution buffer, StageTips containing 2 Empore C18 (3 M) punches were conditioned and equilibrated with 1 × 100 μL methanol (MeOH), 1 × 100 μL 50% ACN/0.1% FA, and 2 × 100 μL 0.1% FA.

After elution of enriched peptides from anti-K-ɛ-GG antibody, samples were spun down for 30 s at 2000 r.c.f. and the supernatant containing the eluted peptides was removed from the beads and added directly to a conditioned StageTip. Extra care was taken to not pipette antibody beads. A second elution of peptides from the antibody beads was performed exactly as described above. During the second elution incubation, the supernatant of the first elution was spun through the conditioned StageTips at 3500 r.c.f. for 2 min. After the second elution incubation was completed, the tubes were spun down for 30 s at 2000 r.c.f., the supernatants were loaded onto the StageTips, and supernatant was spun through at 3500 r.c.f. for 2 min. StageTips were washed with 2 × 100 μL 0.1% FA. Peptides were eluted from StageTips with 1 × 50 μL 50% ACN/0.1% FA and transferred to HPLC vials and dried to completion.

Peptides were reconstituted in 9 μL of 3% MeCN/0.1% FA and analyzed by online nanoflow LC-MS/MS using a Q-Exactive+ mass spectrometer (Thermo Fisher Scientific) coupled online to an Easy-nLC 1000 (Thermo Fisher Scientific). Briefly, 4 μL of sample was loaded onto a microcapillary column (360 μm OD × 75 μm ID) containing an integrated electrospray emitter tip (10 μm) (New Objective) packed with 24 cm of ReproSil-Pur C18-AQ 1.9 μm beads (Dr. Maisch GmbH). The nanoflow column was heated to 50 °C using a column heater (Phoenix S&T). Samples were analyzed using a 110 min LC-MS method. Mobile phase flow rate was 200 nL/min. Solvent A comprised 3% acetonitrile/0.1% FA. Solvent B comprised 90% acetonitrile/0.1% FA. The LC-MS/MS method used the following gradient profile: (min:%B) 0:2; 1:6; 85:30; 94:60; 95:90; 100:90; 101:50; 110:50 (the last two steps at 500 nL/min flow rate). The mass spectrometer was operated such that MS1 spectra were measured with a resolution of 70,000, an AGC target of 3 × 10^6^, and a mass range from 300 to 1800 *m/z*. The top 12 most abundant precursors were triggered for MS/MS at a resolution of 35,000, an AGC target of 5 × 10^4^, an isolation window of 0.7 *m/z*, a maximum ion time of 150 ms, and a normalized collision energy of 30. The dynamic exclusion time was set to 20 s, the peptide match was set to preferred, and charge state screening was enabled to reject precursor charge states that were unassigned, 1, or >6.

### Quenching of on-antibody TMT labeling reactions

To test the feasibility of quenching TMT reactions for on-antibody labeling, replicate 1 mg aliquots of Jurkat peptides were prepared and enriched with anti-K-ɛ-GG antibody exactly as described above. On-antibody TMT labeling was completed using 0.4 mg of a single TMT10 reagent for 10 min exactly as described above. Quenching was performed after the TMT incubation step by adding 8 μL of 5% hydroxylamine to each sample, spinning down quickly at 2000 r.c.f. for 5–10 s, and shaking samples at RT for 5 min at 1400 r.p.m. Sample washing post TMT incubation, peptides elution from the antibody, desalting, and LC-MS/MS analysis were performed exactly as described above for TMT reagent amount and reaction time optimizations.

### Comparison of on-antibody and in-solution TMT labeling

To compare results of on-antibody and in-solution TMT labeling, replicate 1 mg aliquots of Jurkat peptides were enriched and labeling by each respective method.

For on-antibody labeling, Jurkat peptides were prepared, incubated with anti-K-ɛ-GG antibody beads, TMT labeled with 0.4 mg TMT10 reagent for 10 min, and quenched exactly as described above for TMT quenching experiments. After quenching, samples were washed 4 × 1.5 mL ice cold IAP buffer followed by 1 × 1.5 mL ice cold PBS. The final wash buffer was removed and samples were eluted, desalted, and analyzed by LC-MS/MS as described for TMT reagent amount and reaction time optimization.

For in-solution TMT labeling, Jurkat peptides were prepared and incubated with anti-K-ɛ-GG antibody beads exactly as described for TMT reagent amount and reaction time optimization. Following incubation, all samples were centrifuged at 2000 r.c.f. for 1 min and then antibody beads were allowed to settle by letting tubes sit for ~10–20 s on ice. The supernatant (IP flowthrough) was removed and the antibody beads were washed with 3 × 1.5 mL of ice cold IAP buffer and 2 × 1.5 mL ice cold PBS. Samples were then eluted with 0.15% TFA and desalted with StageTips exactly as described for TMT reagent amount and reaction time optimization experiments. After elution from StageTips, peptides were kept in 1.5 mL tubes and dried to completion.

Dried K-ɛ-GG peptides were resuspended in 18 μL of 200 mM HEPES. Four microliters of 100% ACN was added. TMT labeling was performed by adding 3 μL of a TMT10 reagent (0.8 mg/41 μL) to each sample and shaking samples at RT for 1 h at 700 r.p.m. TMT labeling was quenched by adding 2 μL of 5% hydroxylamine to each sample and shaking samples at RT for 15 min at 700 r.p.m. Samples were acidified with 2.7 μL 10% FA and dried to completion.

Dried labeled K-ɛ-GG peptides were resuspended in 150 μL 0.1% FA. StageTips containing 2 Empore C18 (3 M) punches were conditioned and equilibrated with 1 × 100 μL methanol (MeOH), 1 × 100 μL 50% ACN/0.1% FA, and 2 × 100 μL 0.1% FA. The resuspended peptides were loaded onto the StageTips, and spun through at 3500 r.c.f. for 2 min. The StageTips were washed with 2 × 100 μL 0.1% FA. Peptides were eluted with 1× 50 μL 50% ACN/0.1% FA. Eluted peptides were transferred to HPLC vials and dried to completion. LC-MS/MS analysis was performed as described for TMT reagent amount and reaction time optimization.

### On-antibody vs in-solution labeling for a TMT10 experiment

HeLa S3 cells (ATCC CCL-2.2) were cultured in RPMI-1640 media (Invitrogen) supplemented with 5% dialyzed fetal bovine serum (Sigma-Aldrich), penicillin, streptomycin, and glutamine (Invitrogen). HeLa tryptic peptides were generated exactly as described above for Jurkat cells. To generate data shown in Supplementary Fig. 4, two different TMT10-plex experiments were completed, each containing 10 × 1 mg peptide process replicates as input per TMT channel. One TMT10-plex experiment was used to test on-antibody TMT labeling following K-ɛ-GG peptide enrichment and the other plex was used to test in-solution TMT labeling following K-ɛ-GG peptide enrichment.

For the on-antibody TMT10-plex experiment 31.25 μg of crosslinked anti-K-ɛ-GG antibody from 0.5 μg/μL solution was pipetted into 10 × 1.5 mL Eppendorf tubes. Each 1 mg peptide sample was resuspended in 1.5 mL of IAP buffer and shaken at room temperature (RT) for 5 min. The pH of each sample was checked by spotting ~1 μL of solution on pH paper to ensure the pH was ~7. If the pH was acidic, the samples were titrated using 1 M Tris. Peptide samples were then centrifuged at 5000 r.c.f. for 5 min. Each sample was added to its own tube of aliquoted antibody and incubated for 1 hr at 4 °C, with gentle end-over-end rotation. After incubation, all enrichments were kept on ice unless being handled or centrifuged. Following incubation, all samples were centrifuged at 2000 r.c.f. for 1 min and antibody beads were allowed to settle by letting tubes sit for ~10–20 s on ice. The supernatant (IP flowthrough) was removed and the antibody beads were washed with 1.5 mL of ice cold IAP buffer. For washing the beads, after the addition of the wash reagent, the tubes were inverted ~5 times, centrifuged for ~30–60 s at 2000 r.c.f., allowed to sit for ~10–20 s to let the beads settle, and the supernatant was removed. All washes were completed as quickly as possible. A wash with 1.5 mL of ice cold PBS was performed and antibody beads were resuspended in 200 μL of 100 mM HEPES (pH 8.5).

On-antibody TMT labeling was performed by adding 0.4 mg of the appropriate TMT10 reagent (resuspended in 10 μL of ACN) to each enrichment sample. Samples were spun down quickly at 2000 r.c.f. for 5–10 s and incubated at RT for 10 min while shaking at 1400 r.p.m. Quenching of the TMT labeling reaction was performed by adding 8 μL of 5% hydroxylamine to each sample, spinning down quickly at 2000 r.c.f. for 5–10 s, and shaking samples at RT for 5 min at 1400 r.p.m. Following quenching, samples were washed with 1.3 mL ice cold IAP buffer followed by 1.5 mL ice cold IAP buffer. Next, 130 μL of ice cold IAP buffer was added to each tube and used to resuspend the antibody beads. All the antibody beads from each tube were transferred and combined in a new 1.5 mL tube. Combined antibody beads were washed with the IAP buffer used for the bead transfer. The empty tubes previously containing antibody beads were serially washed once with 1.5 mL ice cold IAP buffer. Afterwards, the combined antibody beads were washed with this IAP buffer. Combined antibody beads were washed once with 1.5 mL ice cold PBS.

The final wash buffer was removed and peptides were eluted from the antibody beads by adding 150 μL of 0.15% TFA to the beads forcefully enough to resuspend them and incubating for ~5 min at RT. While the beads were incubating in elution buffer, a StageTip containing 2 Empore C18 (3 M) punches was conditioned and equilibrated with 1 × 100 μL methanol (MeOH), 1 × 100 μL 50% ACN/0.1% FA, and 2 × 100 μL 0.1% FA. After elution of enriched peptide from the anti-K-ɛ-GG antibody, the combined sample was spun down for 30 s at 2000 r.c.f. and the supernatant containing the eluted peptides was removed from the beads and added directly to the conditioned StageTip. Extra care was taken to not pipette antibody beads. A second elution of peptides from the antibody beads was performed exactly as described above. During the second elution incubation, the supernatant of the first elution was spun through the StageTip at 3500 r.c.f. for 2 min. After the second elution incubation was completed, the tube with combined antibody beads was spun down for 30 s at 2000 r.c.f., the supernatant was loaded onto the StageTip, and supernatant was spun through at 3500 r.c.f. for 2 min. The StageTip was washed with 2 × 100 μL 0.1% FA. Peptides were eluted with 1 × 50 μL 50% ACN/0.1% FA. Eluted peptides were transferred to an HPLC vial and dried to completion.

Peptides were reconstituted in 9 μL of 3% MeCN/ 0.1% FA and analyzed by online nanoflow LC-MS/MS using an Easy-nLC1200 coupled to an Orbitrap Fusion Lumos (Thermo Fisher Scientific) mass spectrometer. Briefly, 4 μL of sample was loaded onto a microcapillary column (360 μm OD × 75 μm ID) containing an integrated electrospray emitter tip (10 μm) (New Objective) packed with 24 cm of ReproSil-Pur C18-AQ 1.9 μm beads (Dr. Maisch GmbH). The nanoflow column was heated to 50 °C using a column heater (Phoenix S&T). Samples were analyzed using a 154 min LC-MS method. Mobile phase flow rate was 200 nL/min. Solvent A comprised 3% acetonitrile/0.1% FA. Solvent B comprised 90% acetonitrile/0.1% FA. The LC-MS/MS method used the following gradient profile: (min:%B) 0:2; 1:6; 122:35; 130:60; 133:90; 143:90; 144:50; 154:50 (the last two steps at 500 nl/min flow rate). The mass spectrometer was operated such that MS1 spectra were measured with a resolution of 60,000, an AGC target of 4 × 10^5^, and a mass range from 350 to 1800 *m/z*. A top speed approach (cycle time 2 s) was used to trigger MS/MS at a resolution of 50,000, an AGC target of 1 × 10^5^, an isolation window of 0.7 *m/z*, a maximum ion time of 150 ms, and a normalized collision energy of 38. The dynamic exclusion time was set to 20 s, the peptide match was set to peptide mode, and charge state screening was enabled to reject precursor charge states that were unassigned, 1, or >6.

For the in-solution TMT10-plex experiment, the 10 × 1 mg HeLa peptide samples were prepared and incubated with anti-K-ɛ-GG antibody as described for the on-antibody TMT10-plex above. Following incubation, all 10 samples were centrifuged at 2000 r.c.f. for 1 min and antibody beads were allowed to settle by letting tubes sit for ~10–20 s on ice. The supernatant (IP flowthrough) was removed and the antibody beads were washed with 3 × 1.5 mL ice cold IAP buffer, 2 × 1.5 mL ice cold PBS. After removing the final wash buffer, all 10 samples were eluted with 0.15% TFA acid and individually desalted with StageTips containing 2 Empore C18 (3 M) punches as described above for the on-antibody TMT10-plex method. After elution from StageTips, peptides were kept in 1.5 mL tubes and dried to completion.

Each of the 10 dried K-ɛ-GG peptide samples were resuspended in 18 μL of 200 mM HEPES. Once resuspended, 4 μL of 100% ACN was added to each sample. TMT labeling of each sample was performed by adding 3 μL of the appropriate TMT10 reagent (0.8 mg/41 μL) to each sample followed by shaking at RT for 1 h at 700 r.p.m. TMT labeling was quenched by adding 2 μL of 5% hydroxylamine to each sample and shaking samples at RT for 15 min at 700 r.p.m. All samples were combined into a single tube, acidified by adding 3 μL of 100% FA, and dried to completion.

Dried labeled K-ɛ-GG peptides were resuspended in 150 μL 0.1% FA. A StageTip containing 2 Empore C18 (3 M) punches was conditioned and equilibrated with 1 × 100 μL methanol (MeOH), 1 × 100 μL 50% ACN/0.1% FA, and 2 × 100 μL 0.1% FA. The resuspended peptides were loaded onto the StageTip, and spun through at 3500 r.c.f. for 2 min. The StageTip was washed with 2 × 100 μL 0.1% FA. Peptides were eluted with 1× 50 μL 50% ACN/0.1% FA. Eluted peptides were transferred to HPLC vials and dried to completion. LC-MS/MS analysis was performed using the exact parameters described above for the on-antibody TMT10-plex method.

### Ubiquitylation profiling of lenalidomide-treated cells

MM1S cells (ATCC #2974) were cultured in RPM1-1640 (Invitrogen) supplemented with 10% dialyzed fetal bovine serum (Sigma-Aldrich), penicillin, streptomycin, and glutamine (Invitrogen). Cells were treated for 12 h with 1 μM lenalidomide (Selleck) and for 3 h with 5 μM MG-132 (Selleck) or treated with only 5 μM MG-132 for 3 h. MM1S cells were lysed for 30 min with ice cold urea lysis buffer containing 8 M urea, 75 mM NaCl, 50 mM Tris-HCl (pH 8.0), 1 mM EDTA, 2 μg/mL Aprotinin (Sigma, A6103), 10 μg/mL Leupeptin (Roche #11017101001), 1 mM PMSF (Sigma, 78830), 1 mM CAM (Sigma, 22790), and 50 μM PR-619 (LifeSensors, SI9619). Tryptic peptides were generated exactly as described above for Jurkat cells. For the TMT10-plex experiment shown in Fig. 2, five channels consisted of process replicates from lenalidomide + MG-132-treated samples and the other five channels consisted of process replicates from MG-132-treated samples. An input of 1 mg peptide was used for each experimental condition. K-ɛ-GG peptide enrichment and on-antibody TMT labeling was completed exactly as described above for the TMT10-plex HeLa cell experiment.

The final TMT10 K-ɛ-GG-enriched sample was reconstituted in 14 μL of 3% MeCN/ 0.1% FA and analyzed on a Fusion Lumos mass spectrometer (Thermo Fisher Scientific) coupled online to an Easy-nLC1200 (Thermo Fisher Scientific). One-third of the sample was analyzed by HCD-MS2, another one-third by FAIMS-HCD-MS2, and the final one-third by SPS-MS3.

MS2 data were acquired using the following method: 4 μL of sample was loaded onto a microcapillary column (360 μm OD × 75 μm ID) containing an integrated electrospray emitter tip (10 μm) (New Objective) packed with 24 cm of ReproSil-Pur C18-AQ 1.9 μm beads (Dr. Maisch GmbH). The nanoflow column was heated to 50 °C using a column heater (Phoenix S&T). Samples were analyzed using a 154 min LC-MS method. Mobile phase flow rate was 200 nL/min. Solvent A comprised 3% acetonitrile/0.1% FA. Solvent B comprised 90% acetonitrile/0.1% FA. The LC-MS/MS method used the following gradient profile: (min:%B) 0:2; 1:6; 122:35; 130:60; 133:90; 143:90; 144:50; 154:50 (the last two steps at 500 nl/min flow rate). The mass spectrometer was operated such that MS1 spectra were measured with a resolution of 60,000, an AGC target of 4 × 10^5^, and a mass range from 350 to 1800 * m/z*. A top speed approach (cycle time 2 s) was used to trigger MS/MS at a resolution of 50,000, an AGC target of 5 × 10^4^, an isolation window of 0.7 *m/z*, a maximum ion time of 150 ms, and a normalized collision energy of 34. The dynamic exclusion time was set to 20 s, the peptide match was set to peptide mode, and charge state screening was enabled to reject precursor charge states that were unassigned, 1, or >6.

FAIMS-MS2 data were acquired using the following method: 4 μL of sample was loaded onto the same microcapillary column (360 μm OD × 75 μm ID) that was used to acquire the MS2 and SPS-MS3 data using the same HPLC settings described for the HCD-MS2 method above. The mass spectrometer was operated such that MS1 spectra were measured with a resolution of 60,000, an AGC target of 4 × 10^5^, and a mass range from 300 to 1800 *m/z*. The Thermo FAIMS Pro device was run with default parameters. The FAIMS source was operated in standard resolution mode at 100 °C. FAIMS compensation voltages (CVs) of 40, 60, and 80 V were used with a top10 method to trigger MS/MS at a resolution of 50,000, an AGC target of 5 × 10^4^, an isolation window of 0.7 *m/z*, a maximum ion time of 150 ms, and a normalized collision energy of 34. The dynamic exclusion time was set to 20 s, the peptide match was set to peptide mode, and charge state screening was enabled to reject precursor charge states that were unassigned, 1, or >6.

SPS-MS3 data were acquired using the following method: 4 μL of sample was loaded onto the same microcapillary column (360 μm OD × 75 μm ID) that was used to acquire the MS2 and FAIMS-MS2 data using the same HPLC settings described for the HCD-MS2 method above. The mass spectrometer was operated such that MS1 spectra were measured with a resolution of 60,000, an AGC target of 4 × 10^5^, and a mass range from 300 to 1800 *m/z*. The top 10 most abundant ions from the MS1 scan were method to trigger MS/MS in the Orbitrap at a resolution of 15,000, an AGC target of 5 × 10^4^, a maximum ion time of 300 ms, and an isolation width of 0.5, using a normalized CID collision energy of 35%. A synchronous-precursor-selection (SPS) MS3 scan was collected using 10 notches. SPS-MS3 precursors were fragmented by HCD and analyzed using the Orbitrap (NCE = 65%, AGC = 100,000, maximum injection time = 500 ms, and resolution = 50K).

### Patient-derived xenografts (PDX) analysis

Proteomic analysis of Breast cancer PDX models, WHIM2, and WHIM16 has been described in detail elsewhere^30^. Briefly, tumor chunks were snap frozen and cryopulverized. Roughly, 100 mg of wet-weight was lysed in Urea lysis buffer as described above followed by digestion using Trypsin and LysC and peptide purification using Sep-Pak cartridge (Waters).

For ubiquitylome analysis, the TMT10-plex experiment shown in Fig. 3, five channels consisted of process replicates from WHIM2 and the other five channels consisted of process replicates from WHIM16. An input of 500 μg peptide was used for each experimental condition. K-ɛ-GG peptide enrichment and on-antibody TMT labeling were completed exactly as described above for the TMT10-plex HeLa cell experiment. The final TMT10 K-ɛ-GG-enriched sample was reconstituted 9 μL of 3% MeCN/ 0.1% FA and analyzed on a Fusion Lumos mass spectrometer (Thermo Fisher Scientific). The sample was analyzed by FAIMS-HCD-MS2 as described for the lenalidomide-treated MM1S sample above. The sample was analyzed using replicate 4 μL injections where only the FAIMS compensation voltages (40, 60, 80 V) and (40, 50, and 70 V), were changed between injections.

For the proteome and acetylome analysis, a total of 300 μg of peptides were labeled using 300ug of TMT reagent using the reduced TMT labeling protocol described before^31^. TMT10-plex experiment consisted of five replicates each of WHIM2 and WHIM16. TMT-labeled peptides were further quenched using 5% hydroxylamine following by combining peptides at equal proportions. TMT label peptides were further purified using Sep-Pak cartridge. Dried peptides were resuspended in 5 mM ammonium formate and fractionated on a high pH reverse phase chromatography unit (Zorbax 300 Extend-C18 column (3.5 μM, 250 mm; Agilent) coupled to Agilent 1260 offline HPLC). A total of 72 fractions were concatenated into a total of 24 fractions, 5% of which was dried down for proteome analysis. For proteome analysis, 500 ng peptides per fraction was injected on a Proxeon nLC1200-Thermo Lumos instrument setup running on a 110 min method at 200 nl/min flow rate. Details of the LC and MS parameters can be found in ref. ^30^. In brief, the 110-min LC-MS/MS method consisted of a 10-min column-equilibration procedure; a 20 min sample-loading procedure; and the following gradient profile: (min:%B) 0:2; 1:6; 85:30; 94:60; 95;90; 100:90; 101:50; 110:50 and the last two steps were at 500 nL/min flow rate. The MS parameters are as follows: MS1—resolution: 60,000, AGC target: 4E5, mass range: 350–1800 *m*/*z*, injection time: 50 ms. MS2—resolution: 50,000, AGC target: 6E4, injection time: 110 ms, isolation window: 0.7 *m*/*z*. Data-dependent mode cycle time was set to 2 s.

The residual 95% from each of 24 fractions were further concatenated. Every fourth fraction was pooled together yielding a total of four fractions. Dried down fractions were dissolved in 1.4 mL of 1× IAP buffer and were subjected to acetyl-peptide enrichment using acetyl-lysine enrichment kit from Cell Signaling Technologies. A single vial was used for the entire plex which is a total of 10 μL beads per fraction. Peptide-antibody incubation was performed at 4 °C for 2 h, followed by four washes using ice cold PBS. Peptides were eluted using 0.15% TFA and purified using C18 StageTips. Acetylated peptides were analyzed using a Proxeon nLC1200-Thermo Lumos instrument setup running on a 260 min gradient at 200 nL/min flow rate. For acetylome and proteome analysis, the same LC and column setup was used as the proteome, but the gradient was extended to 260 min with the following gradient profile: (min:%B) 0:2; 1:6; 235:30; 244:60; 245;90; 250:90; 251:50; 260:50 and the last two steps were at 500 nL/min flow rate. The MS parameters were identical to MS parameters used for proteome analysis except for MS1 and MS2 injection times of 50 and 125 ms, respectively.

### Data analysis

All data were analyzed using Spectrum Mill software package v 6.1 prerelease (Agilent Technologies). For HCD-MS2, FAIMS-HCD-MS2, and SPS-MS3 data, similar MS/MS spectra acquired on the same precursor *m/z* ±1.4 within ±45 s were merged. For lenalidomide-treated MM1S data, MS/MS spectra acquired on the same precursor *m/z* ±1.4 within ±45 s were merged based on precursor selection purity and spectral similarity. For SPS-MS3 data, CID-MS2 and HCD-MS3 scans were merged from the same precursor. MS/MS spectra were excluded from searching if they were not within the precursor MH+ range of 750–6000 Da, or if they failed the quality filter by not having a sequence tag length >0. MS/MS spectra were searched against a UniProt database containing 59,079 human proteins. The databases were downloaded from the UniProt website and redundant sequences were removed; and a set of common laboratory contaminant proteins (150 sequences) was appended. All spectra were allowed ±20 ppm mass tolerance for precursor and product ions, 30% minimum matched peak intensity, and “trypsin allow P” enzyme specificity with up to four missed cleavages. The instrument setting was ESI Q Exactive HCD for MS2 spectra and ESI Orbitrap for SPS-MS3 spectra. Fragmentation mode was all for MS2 spectra, and CID for SPS-MS3 spectra. The fixed modifications were carbamidomethylation at cysteine for all data, and TMT10 partial-mix (N-term, K) for ubiquitylation data, and TMT10 full Lys only-mix (N-term, K) for acetylation and proteome data. For ubiquitylation data, allowed variable modifications were Ubiquitin Residual GG from Tryp Cut on K, oxidized methionine, and acetylation of protein N termini with a precursor MH+ shift range of −134 to 375 Da. A target–decoy FDR of 1.2% was used for ubiquitin site data. For acetylated peptide data, allowed variable modifications were N-terminal acetylation, acetylated lysine, oxidized methionine, N-terminal pyroglutamic acid, and deamidated asparagine. The precursor mass shift used was −400 to 70 Da. For proteome searches, the above variable modifications were used except for acetylated lysine and precursor mass shift used used was −18 to 64.

PDX proteome, acetylome, and ubiquitylome data were analyzed using RefSeq database containing 37,592 human and 27,289 mouse entries and complemented with common contaminants (RefSeq.20160914_Human_Mouse_ucsc_hg19_mm10_customProDBnr_mito_150contams). Spectrum Mill sub-group specific protein grouping feature was used to dissect human and mouse proteins. Details have been described previously^40^. The relative abundance of a protein or peptide was reported as determined using median of TMT reporter intensity from all PSMs corresponding to the protein or peptides. TMT ratio was obtained using multi-median approach described before^30^ where TMT intensity for a corresponding channel was divided by median intensity across all channels. log2 TMT ratios were further normalized by median centering.

For data shown in Supplementary Fig. 4e, mass spectra were also analyzed with MaxQuant software version 1.6.0.16 using a human UniProt database to determine the charge state distribution for the total MS1 precursor intensity for each experiment. MS/MS searches were analyzed using oxidation of methionine, protein N-terminal acetylation, and di-glycine remnant on lysine as variable modifications and carbamidomethylation as a fixed modification. Trypsin/P was selected as the digestion enzyme with a maximum of two missed cleavages per peptide. The peptide mass tolerance was set to 20 ppm for the first search and 4.5 ppm for the main search. The FTMS MS/MS mass tolerance was set to 20 ppm. PSM and protein FDRs were 1% for identification. The allPeptides table was used to determine the charge state distribution of the total MS1 precursor intensity in all the experiments.

For ubiquitylome data generated from lenalidomide-treated MM1S cells (data shown in Fig. 2 and Supplementary Fig. 5), TMT ratios were obtained using multi-median approach where TMT intensity for a corresponding channel was divided by median intensity across all channels. Log2 TMT ratios were further normalized by median centering. We used an in-house application, Protigy (https://github.com/broadinstitute/protigy), to perform statistical data analysis. Differential K-ɛ-GG sites between lenalidomide-treated and non-lenalidomide-treated MM1S cells were identified using a two-tailed two-sample moderated *t*-test using the limma R-package^41^ implemented in Protigy. *P* values were adjusted for multiple hypothesis testing using the Benjamini–Hochberg method.

For ubiquitylome data generated from Breast cancer PDX models (data shown in Fig. 3), we identified differential K-ɛ-GG sites between WHIM2 and WHIM16 cells using a two-tailed two-sample moderated *t*-test as described for lenalidomide-treated MM1S cells.

Pathway enrichment was performed using DAVID ontology enrichment tool^42^.

### Reporting summary

Further information on research design is available in the Nature Research Reporting Summary linked to this article.

## Supplementary information

Supplementary Information

Description of Additional Supplementary Files

Supplementary Data 1

Supplementary Data 2

Supplementary Data 3

Supplementary Data 4

Supplementary Data 5

Supplementary Data 6

Supplementary Data 7

Reporting Summary

## Data Availability

The original mass spectra and the protein sequence databases used for searches have been deposited in the public proteomics repository MassIVE (http://massive.ucsd.edu) and are accessible at ftp://massive.ucsd.edu/MSV000084650/. The source data underlying Fig. 3b, Supplementary Figs. 1a, c, 3b, and 4d can be found in Supplementary Data 7a, Supplementary Data 1, Supplementary Data 2, Supplementary Data 4, Supplementary Data 5a, b, respectively. All other data are available from the corresponding authors on reasonable request.
