# Supplementary material for: Rapid and deep-scale ubiquitylation profiling for biology and translational research
Source: Nat Commun. 2020 Jan 17;11:359. doi: 10.1038/s41467-019-14175-1 (PMC6969155; doi:10.1038/s41467-019-14175-1)
Supplement: Supplementary file 1 — Supplementary Information [file 41467_2019_14175_MOESM1_ESM.pdf]

Supplementary Information

Rapid and deep-scale ubiquitylation profiling for biology and translational research

Namrata D. Udeshi[1*#], Deepak C. Mani[1*], Shankha Satpathy[1], Shaunt Fereshetian[1], Jessica A. Gasser[1,2,3], Tanya Svinkina[1], Meagan E. Olive[1], Benjamin L. Ebert[1,2,3,4], Philipp Mertins[1,5,6], Steven A. Carr[1#]

*These authors contributed equally to this work

# corresponding authors NDU: udeshi@broadinstitute.org SAC: scarr@broad.mit.edu

1 Broad Institute of MIT and Harvard, Cambridge, MA, 02142, USA

2 Division of Hematology, Brigham and Women's Hospital, Boston, MA 02115, USA

3 Department of Medical Oncology, Dana-Farber Cancer Institute, Boston, MA 02215, USA

4 Howard Hughes Medical Institute, Dana-Farber Cancer Institute, Boston, MA 02215, USA

5 Max Delbrück Center for Molecular Medicine in the Helmholtz Society, Berlin, Germany

6 Berlin Institute of Health, Berlin, Germany

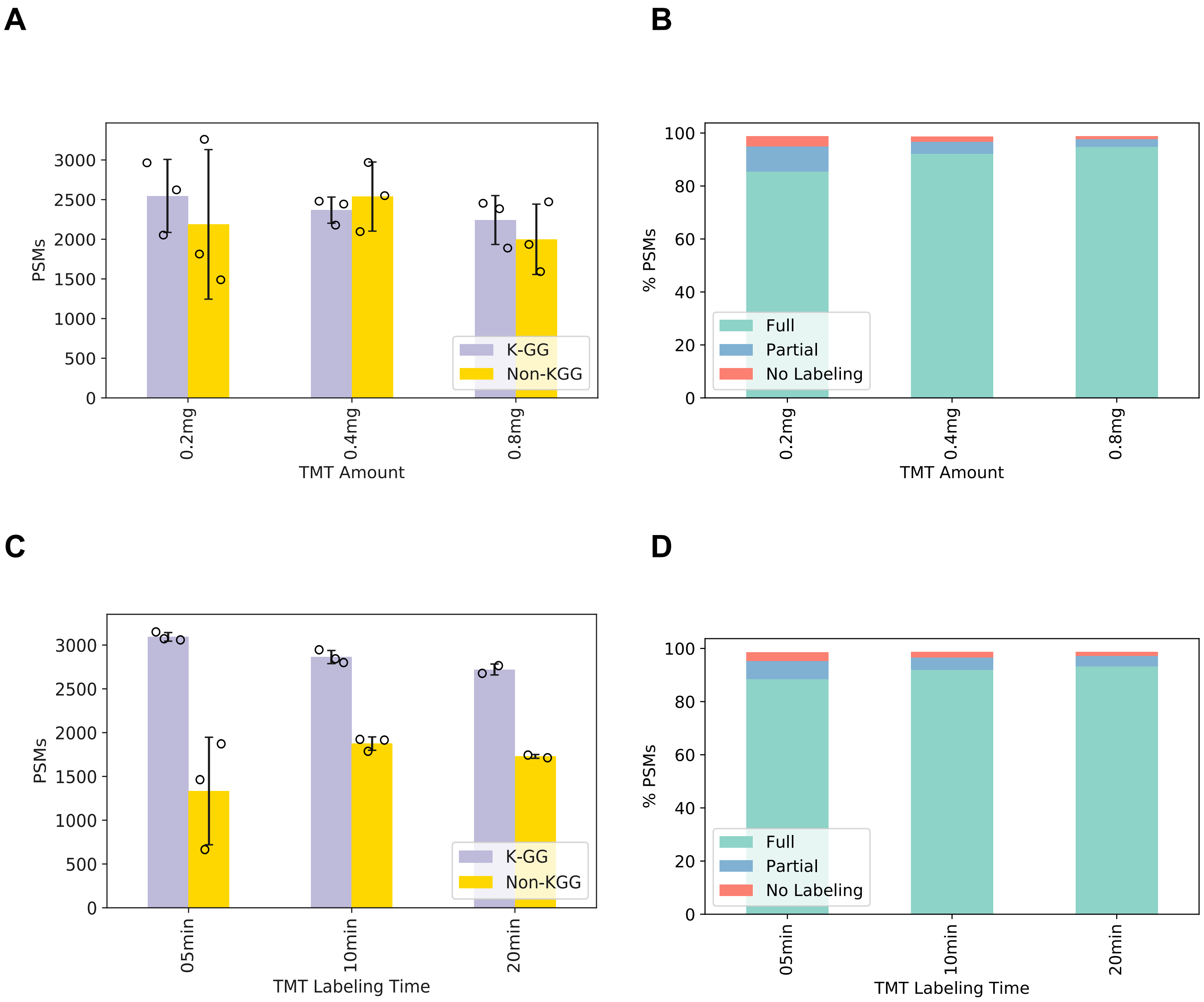

Supplementary Figure 1 Optimization of on-antibody TMT labeling reagent amount and labeling time. 1 mg Jurkat peptide was processed and labeled (n=3) in triplicate using on-antibody TMT labeling with a single TMT reagent under the varying conditions shown. The antibody beads were washed to remove non-specifically bound peptides and excess reagent. Bound peptides were then eluted and analyzed by LC-MS/MS using a 110 min gradient. A) Bar plots show the number of PSMs identified using varying amounts of TMT reagent. Error bars indicate standard deviation across three replicates. Source Data is provided in Supplementary Data 1. B) Stacked bar plots show the % of K-ε-GG PSMs fully, partially, or not labeled by TMT for each TMT reagent amount shown. C) Bar plots show the number of distinct K-ε-GG PSMs identified for each TMT labeling time (min). Error bars indicate standard deviation across three replicates, except for the 20 min time point for which duplicate measurements were completed. Source Data is provided in Supplementary Data 2. D) Stacked bar plots show the % of K-ε-GG PSMs fully, partially, or not labeled by TMT for each labeling time (min). K-ε-GG peptides were considered partially labeled if any, but not all, primary amine (i.e. free N-terminus of the peptide and/or side-chain of Lys), other than the K-ε-GG site itself, was labeled by TMT. A peptide was considered to be fully-labeled if all possible primary amines in the peptide (i.e. N-terminus of the peptide and side-chain of Lys), other than the K-ε-GG site itself, were labeled by TMT.

TMT Labeling Conditions	Distinct K-ε-GG PSMs	Partially Labeled K-ε-GG PSMs	Fully Labeled K-ε-GG PSMs	Relative Yield of K-ε-GG PSMs[1]
10 min, 0.4 mg TMT Labeling	5579	97.4%	92.6%	60.6%
10 min, 0.4 mg TMT Labeling with 5% hydroxylamine	6145	96.9%	90.8%	63.8%
Label Free	5438			47.9%

1 Relative yield of ubiquitylated peptides versus non-ubiquitylated peptides in the samples analyzed

Supplementary Figure 2 Quenching with hydroxylamine helps prevent TMT cross-labeling using on-antibody labeling. 1 mg Jurkat peptide was processed and labeled (n=3) using on-antibody TMT labeling under the varying conditions shown. The antibody beads were washed to remove non-specifically bound peptides and excess reagent. Bound peptides were then eluted and analyzed by LC-MS/MS using a 110 min gradient. Quenching also increased the number of K-e-GG peptides identified.

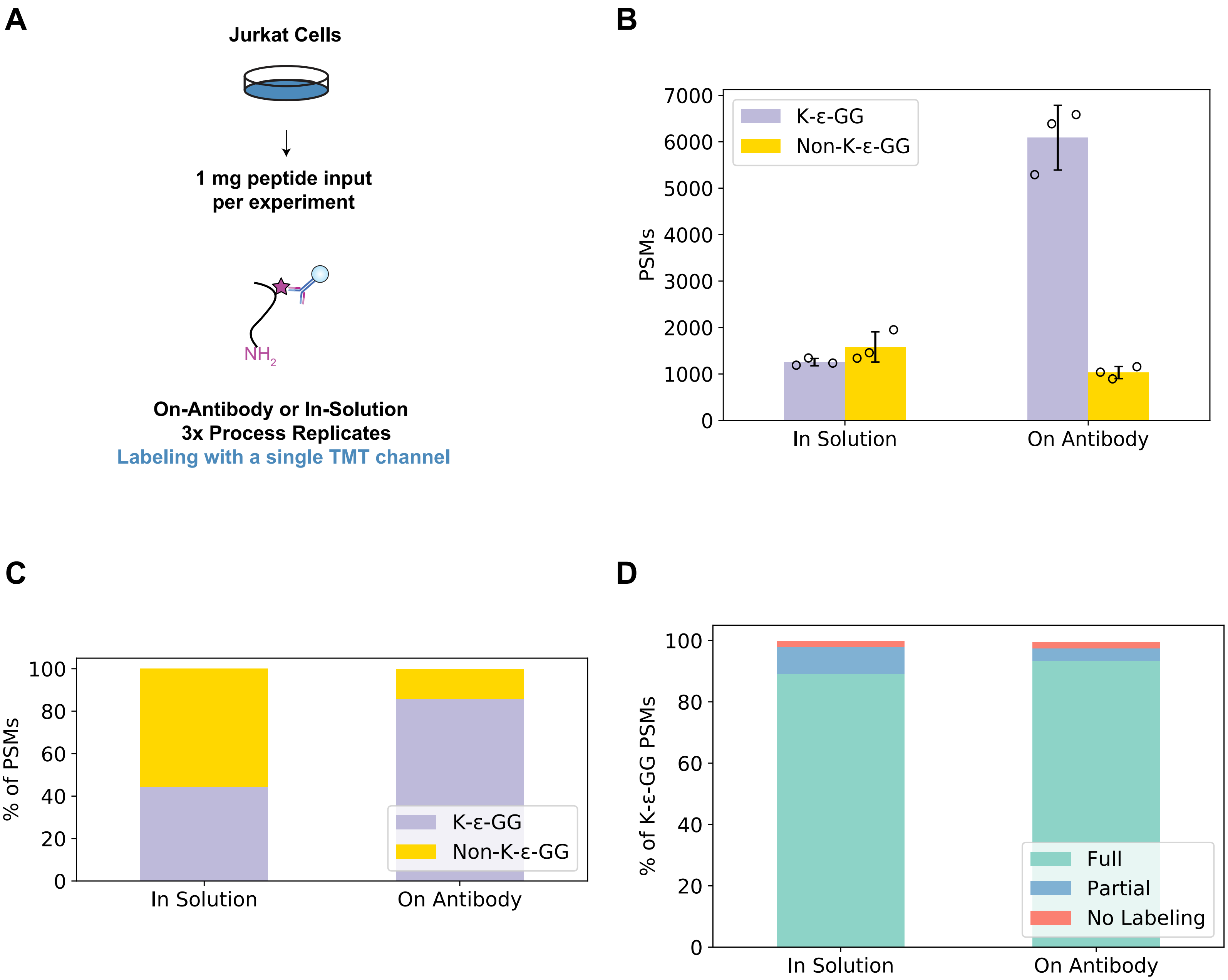

Supplementary Figure 3 Comparison of numbers of identified K-ε-GG and non-K-ε-GG peptides using on-antibody vs. in solution TMT labeling. A) 1 mg Jurkat peptide was enriched and labeled (n=3) with a single TMT channel using the on-antibody or in-solution TMT labeling method. Each replicate was analyzed by LC-MS/MS using a 110 min gradient. B) Bar plots show the number of K-ε-GG and non-K-ε-GG PSMs for in-solution and on-antibody TMT labeling methods. Error bars indicate standard deviation across three replicates. Source data is provided in Supplementary Data 4. C) Stacked bar plots show the % of K-ε-GG and non-K-ε-GG PSMs identified by each TMT labeling method. D) Stacked bar plots show the % of K-ε-GG PSMs fully, partially, or not labeled by TMT for each TMT method. K-ε-GG peptides were considered partially labeled if any, but not all, primary amine (i.e. free N-terminus of the peptide and/or side-chain of Lys), other than the K-ε-GG site itself, was labeled by TMT. A peptide was considered to be fully-labeled if all possible primary amines in the peptide (i.e. N-terminus of the peptide and side-chain of Lys), other than the K-ε-GG site itself, were labeled by TMT. The yield of fully TMT labeled peptides was 4% higher using on-antibody labeling versus in-solution labeling.

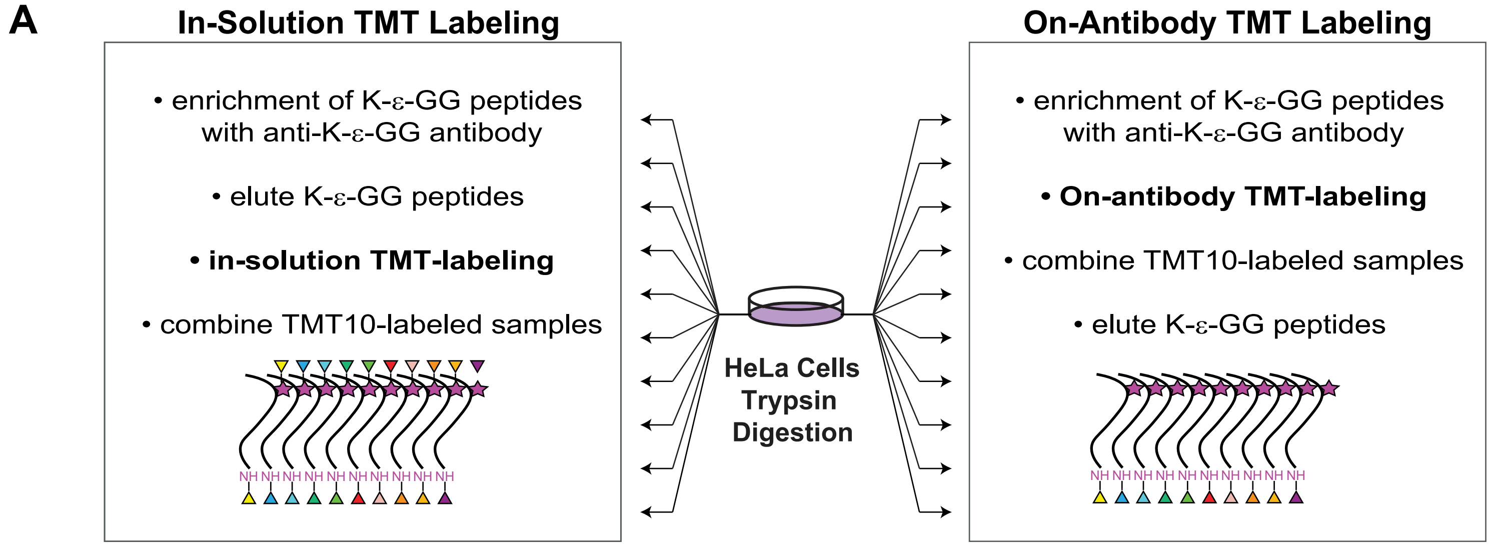

B

Method	Total Distinct K-ε-GG Peptides	Fully Quantified Distinct K-ε-GG Peptides	Fully Quantified Localized Distinct K-ε-GG Peptides	Relative Yield of K-ε-GG Peptides	# of Replicate Injections	Total Analysis Time
On-antibody	9628	9069	8971	85.4%	2	5.1 hr
In-solution	4696	4587	4543	49.9%	2	5.1 hr

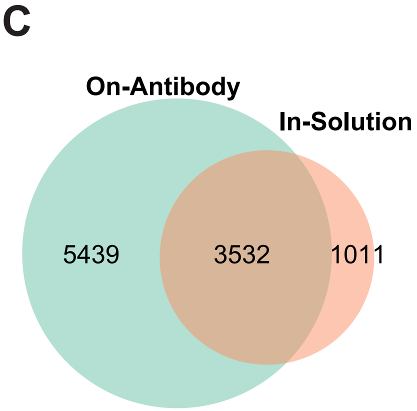

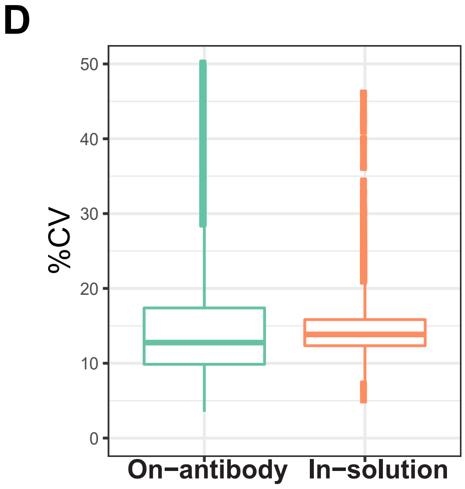

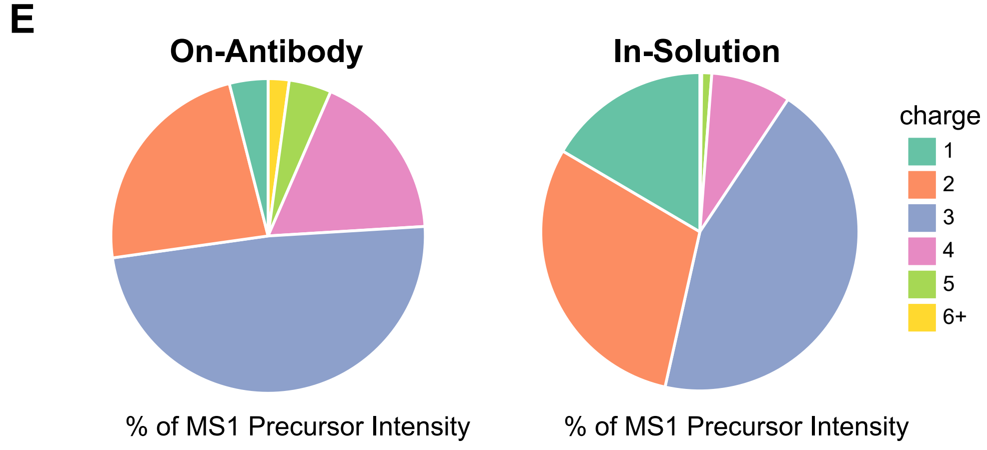

Supplementary Figure 4. Comparison of on-antibody and in-solution labeling methods A) Experimental design for comparison of TMT10 labeling of ubiquitylated peptides enriched from 1 mg HeLa cells/sample. Labeling was done using the on-antibody method and the in-solution method B) Results of ubiquitylome analyses using the on-antibody and in-solution labeling methods; approximately twice the number of ubiquitylated peptides were obtained using the on-antibody labeling method vs. the in-solution approach. C) Overlap in identified fully quantified, and localized distinct K-ε-GG peptides obtained using the on-antibody and in-solution labeling methods D) Reproducibility (% Coefficients of variation) between HeLa process replicates (n=10) for each method. Boxplots depict upper and lower quaritles, with the median shown as a solid line. Whiskers show 1.5 interquartile range and points show outliers. Source Data is provided in Supplementary Data 5A,B E) % of total precursor intensities separated by charge for each method. Samples were analyzed using a 154 min LC-MS/MS method.

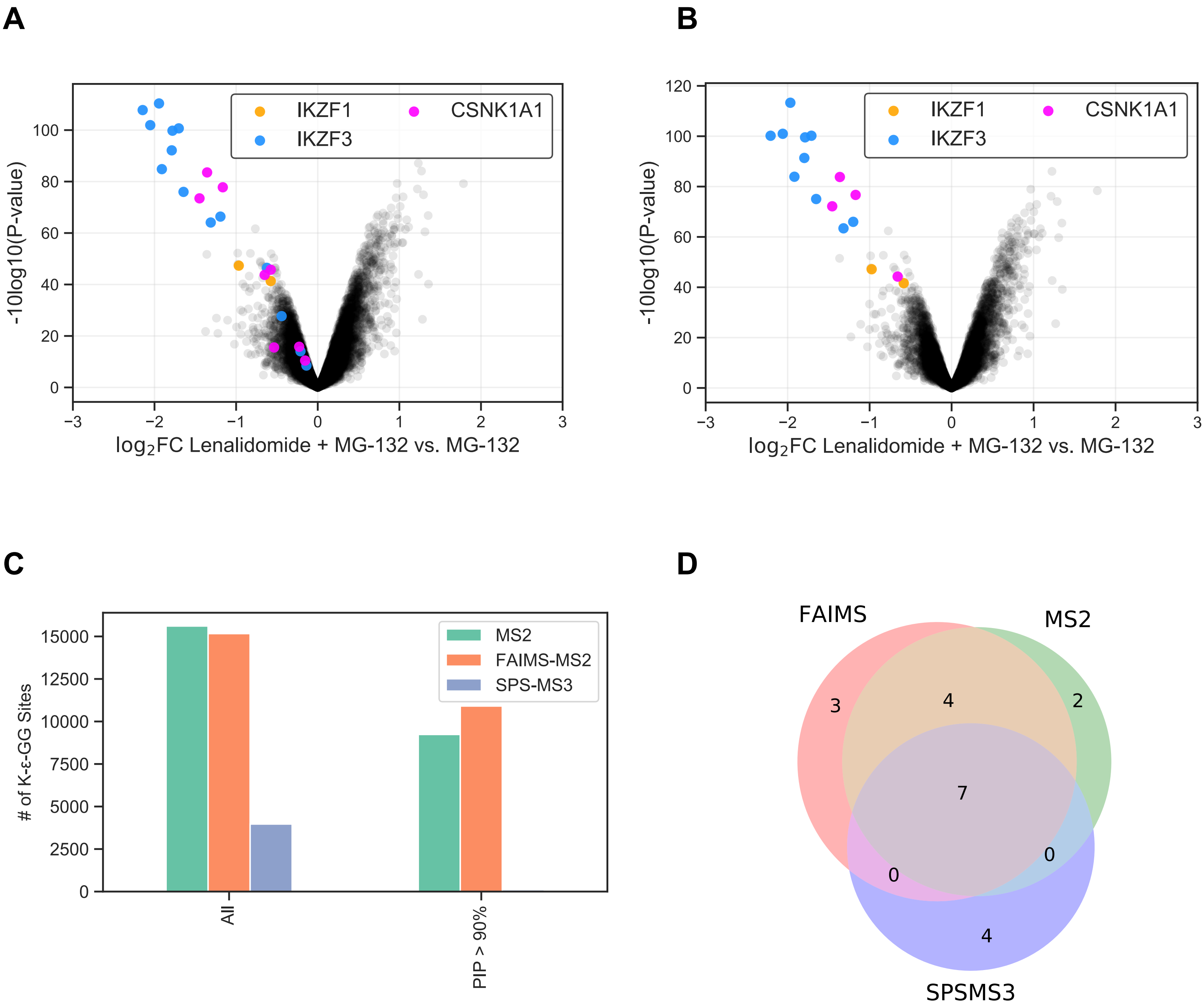

Supplementary Figure 5 Comparison of MS2, FAIMS-MS2, and SPS-MS3 for the analysis the ubiquitylome of lenalidomide-treated MM1S cells. A) Volcano plot of K-ε-GG sites quantified using MS2; fold change is plotted versus the −log10 of their Padj values following a moderated two-sample t-test. K-ε-GG sites from IKZF1, IKZF3, and CSNK1A1 are colored as indicated in the figure legend B) Volcano plot of K-ε-GG sites quantified using MS2 and filtered for a precursor ion purity (PIP) value of ≥ 90%; fold change is plotted versus the −log10 of their Padj values following a moderated two-sample t-test. C) Bar plots show the number of K-ε-GG sites quantified for each MS method. The second group of bars (right) shows the number of K-ε-GG sites quantified for MS2 and FAIMS-MS2 methods after application of a stringent PIP filter (≥ 90%). D) Venn diagram of the overlap of significant (adj.Pval ≤ 0.001) K-ε-GG sites following a moderated two-sample t-test from IKZF1, IKZF3, and CSNK1A1 across each MS experiment type. For this analysis MS2 and FAIMS-MS2 data were filtered using PIP values ≥ 90%.

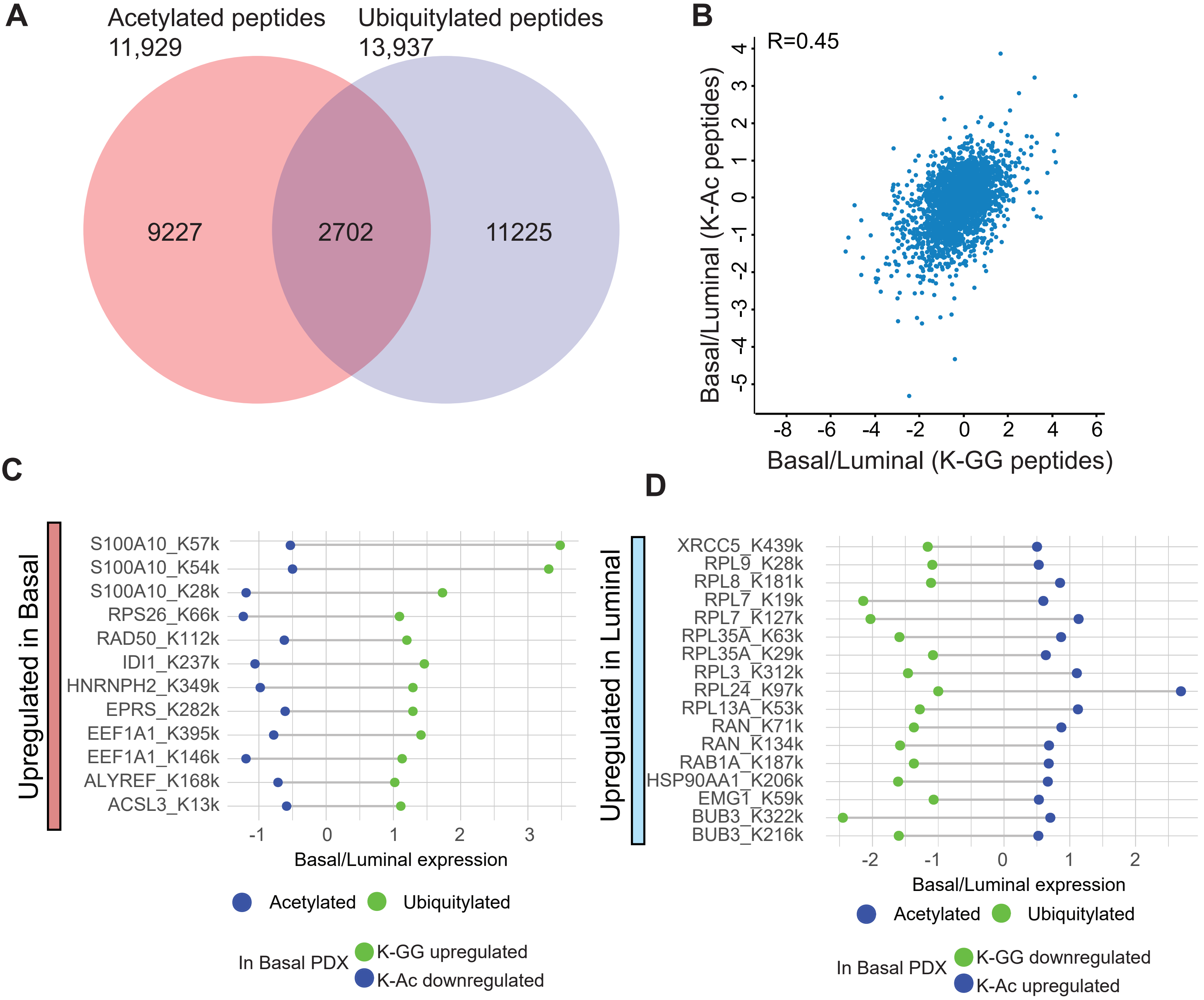

Supplementary Figure 6 Crosstalk between acetylation and ubiquitylation observed in analyses of Luminal and Basal breast cancer patient-derived xenograft (PDX) samples (see Figure 3 main text). A) Venn diagram showing the overlap between acetylated and ubiquitylated sites. B) Scatter plot showing log2 fold-change between Basal and Luminal PDXs, for expression of acetylated and ubiquitylated sites. C,D) Lolipop plots showing log2 fold-change of acetylated and ubiquitylated sites between basal and luminal PDXs. Panel C shows specific sites that were upregulated in ubiquitylation and downregulated in acetylation in the basal vs. luminal PDX samples. Panel D shows specific sites that were downregulated in ubiquitylation and upregulated in acetylation.
