# Supplementary material for: Rapid and deep-scale ubiquitylation profiling for biology and translational research
Source: Nat Commun. 2020 Jan 17;11:359. doi: 10.1038/s41467-019-14175-1 (PMC6969155; doi:10.1038/s41467-019-14175-1)
Supplement: Supplementary file 2 — Description of Additional Supplementary Files [file 41467_2019_14175_MOESM2_ESM.docx]

**Description of Supplementary Files**

**File Name: Supplementary Data 1**

**Description:** Peptide data from TMT labeling amount experiments

**File Name: Supplementary Data 2**

**Description:** Peptide data from TMT labeling time experiments

**File Name: Supplementary Data 3**

**Description:** Peptide data from quenching and label free experiments

**File Name: Supplementary Data 4**

**Description:** On-antibody vs In-solution experiments

**File Name: Supplementary Data 5**

**Description:** On-antibody vs In-solution TMT10 labeling experiments

**File Name: Supplementary Data 6**

**Description:** +/- Lenalidomide MM1S cell data using MS2, FAIMS-MS2, SPS-MS3 methods

**File Name: Supplementary Data 7**

**Description:** PDX ubiquitylome, acetylome and proteome data
