# Supplementary material for: Rapid and deep-scale ubiquitylation profiling for biology and translational research
Source: Nat Commun. 2020 Jan 17;11:359. doi: 10.1038/s41467-019-14175-1 (PMC6969155; doi:10.1038/s41467-019-14175-1)
Supplement: Supplementary file 10 — Reporting Summary [file 41467_2019_14175_MOESM10_ESM.pdf]

natureresearch

Corresponding author(s): Steven Carr and Namrata Udeshi

Last updated by author(s): Dec 18, 2019

Reporting Summary

Nature Research wishes to improve the reproducibility of the work that we publish. This form provides structure for consistency and transparency in reporting. For further information on Nature Research policies, see Authors & Referees and the Editorial Policy Checklist .

Statistics

For all statistical analyses, confirm that the following items are present in the figure legend, table legend, main text, or Methods section.

n/a	Confirmed	
☐	☒	The exact sample size (*n*) for each experimental group/condition, given as a discrete number and unit of measurement
☐	☒	A statement on whether measurements were taken from distinct samples or whether the same sample was measured repeatedly
☐	☒	The statistical test(s) used AND whether they are one- or two-sided<br>*Only common tests should be described solely by name; describe more complex techniques in the Methods section.*
☒	☐	A description of all covariates tested
☒	☐	A description of any assumptions or corrections, such as tests of normality and adjustment for multiple comparisons
☐	☒	A full description of the statistical parameters including central tendency (e.g. means) or other basic estimates (e.g. regression coefficient) AND variation (e.g. standard deviation) or associated estimates of uncertainty (e.g. confidence intervals)
☐	☒	For null hypothesis testing, the test statistic (e.g. *F*, *t*, *r*) with confidence intervals, effect sizes, degrees of freedom and *P* value noted<br>*Give P values as exact values whenever suitable.*
☒	☐	For Bayesian analysis, information on the choice of priors and Markov chain Monte Carlo settings
☒	☐	For hierarchical and complex designs, identification of the appropriate level for tests and full reporting of outcomes
☒	☐	Estimates of effect sizes (e.g. Cohen's *d*, Pearson's *r*), indicating how they were calculated

Our web collection on statistics for biologists contains articles on many of the points above.

Software and code

Policy information about availability of computer code

Data collection	Thermo Xcalibur versions 2.8 and 3.1
Data analysis	Microsoft Excel 2016, Python, R, Spectrum Mill v6 .1, MaxQuant version 1.6.0.16

For manuscripts utilizing custom algorithms or software that are central to the research but not yet described in published literature, software must be made available to editors/reviewers. We strongly encourage code deposition in a community repository (e.g. GitHub). See the Nature Research guidelines for submitting code & software for further information.

Data

Policy information about availability of data

All manuscripts must include a data availability statement. This statement should provide the following information, where applicable:

- Accession codes, unique identifiers, or web links for publicly available datasets
- A list of figures that have associated raw data
- A description of any restrictions on data availability

The original mass spectra and the protein sequence databases used for searches have been deposited in the public proteomics repository MassIVE (http://massive.ucsd.edu) and are accessible at ftp://massive.ucsd.edu/MSV000084650/ . All other data are available from the corresponding authors on reasonable request. The source data underlying Figure 3B, Supplementary Figures 1A, 1C, 3B, and 4D can be found in Supplementary Data 7A, Supplementary Data 1, Supplementary Data 2, Supplementary Data 4, Supplementary Data 5A,5B, respectively.

Field-specific reporting

Please select the one below that is the best fit for your research. If you are not sure, read the appropriate sections before making your selection.

☒ Life sciences ☐ Behavioural & social sciences ☐ Ecological, evolutionary & environmental sciences

For a reference copy of the document with all sections, see nature.com/documents/nr-reporting-summary-flat.pdf

Life sciences study design

All studies must disclose on these points even when the disclosure is negative.

Sample size	No statistical testing was performed to determine sample size.
Data exclusions	MS/MS spectra were excluded from searching in Spectrum Mill if they were not within the precursor MH+ range of 750–6,000 Da, or if they failed the quality filter by not having a sequence tag length >0.
Replication	Replication was successful
Randomization	Randomization was not required in this work due to multiplexing
Blinding	Blinding was not performed int his study

Reporting for specific materials, systems and methods

We require information from authors about some types of materials, experimental systems and methods used in many studies. Here, indicate whether each material, system or method listed is relevant to your study. If you are not sure if a list item applies to your research, read the appropriate section before selecting a response.

Materials & experimental systems

n/a	Involved in the study	
☐	☒	Antibodies
☐	☒	Eukaryotic cell lines
☒	☐	Palaeontology
☐	☒	Animals and other organisms
☒	☐	Human research participants
☒	☐	Clinical data

Methods

n/a	Involved in the study	
☒	☐	ChIP-seq
☒	☐	Flow cytometry
☒	☐	MRI-based neuroimaging

Antibodies

Antibodies used	PTMScan ubiquitin remnant motif (K-ε-GG) kit (Cell Signaling Technology, Kit #5562)
Validation	Validation performed by detection of K-GG peptides after enrichment by LC-MS/MS

Eukaryotic cell lines

Policy information about cell lines

Cell line source(s)	ATCC, USA
Authentication	No authentication was performed
Mycoplasma contamination	Cell line tested negative for mycoplama contamination
Commonly misidentified lines (See ICLAC register)	Hela cells were used to assess technical reproducibility for the methods.

Animals and other organisms

Policy information about studies involving animals; ARRIVE guidelines recommended for reporting animal research

Laboratory animals	8-week-old NOD.Cg-Prkdcscid Il2rgtm1Wjl/SzJ mice (Jackson Laboratories, strain code 005557).

Wild animals	Study did not involve wild animals
Field-collected samples	Study did not involve samples collected from the field
Ethics oversight	PDX models used in this study were approved by the institutional animal care and use committee at Washington University in St. Louis.

Note that full information on the approval of the study protocol must also be provided in the manuscript.
